# IL-2 promotes expansion and intratumoral accumulation of tumor infiltrating dendritic cells in pancreatic cancer

**DOI:** 10.1007/s00262-024-03669-7

**Published:** 2024-03-30

**Authors:** Tingting Gong, Xinyang Huang, Zhuoxin Wang, Ye Chu, Lifu Wang, Qi Wang

**Affiliations:** Department of Gastroenterology, Ruijin Hospital, Shanghai Jiao Tong University School of Medicine, 197 Ruijin Er Road, Shanghai, 200025 China

**Keywords:** Pancreatic adenocarcinoma, interleukin-2, Tumor vaccine, Immunotherapy

## Abstract

This study aims to investigate the diagnostic potential of IL-2 for PDAC and develop a method to improve the dendritic cell (DC) based vaccine against PDAC. The gene expression data and clinical characteristics information for 178 patients with PDAC were obtained from The Cancer Genome Atlas (TCGA). DCs were isolated from Human peripheral blood mononuclear cells (PBMCs) and were cultured in 4 different conditions. DCs were pulsed by tumor cell lysates or KRAS G12D_1 − 23_ peptide, and then used to activate T cells. The mixture of DCs and T cells were administered to xenograft mouse model through the tail vein. The infiltration of DCs and T cells were detected by immunohistochemistry. The generation of KRAS G12D mutation specific cytotoxic T cells was determined by in vitro killing assay. We observed that PDAC patients with higher IL-2 mRNA levels exhibited improved overall survival and increased infiltration of CD8 + T cells, NK cells, naïve B cells, and resting myeloid DCs in the tumor microenvironment. IL-2 alone did not enhance DC proliferation, antigen uptake, or apoptosis inhibition unless co-cultured with PBMCs. DCs co-cultured with PBMCs in IL-2-containing medium demonstrated the strongest tumor repression effect in vitro and in vivo. Compared to DCs obtained through the traditional method (cultured in medium containing GM-CSF and IL-4), DCs cultured with PBMCs, and IL-2 exhibited increased tumor infiltration capacity, potentially facilitating sustained T cell immunity. DCs cultured in the PBMCs-IL-2 condition could promote the generation of cytotoxic T cells targeting tumor cells carrying KRAS G12D mutation.

**Supplementary Information:**

The online version contains supplementary material available at 10.1007/s00262-024-03669-7.

## Introduction

Pancreatic ductal adenocarcinoma is one of the leading causes of cancer related mortality worldwide, with the pancreatic ductal adenocarcinoma (PDAC) as the most common form [1]. The 5-year survival rate of patients with PDAC is 12% and is anticipated to remain dismal for years [2]. The average survival time for PDAC is less than six months if left untreated [3]. At present, surgical removal remains the most effective method for PDAC treatment. However, most of the patients were diagnosed at advanced stage and fewer than 20% PDAC is surgically resectable [4]. The effects of other therapies, such as combination chemotherapy and radiation, are limited because of relapse and resistance [5, 6]. Meanwhile, PDAC is categorized as “cold tumor” which is found to be insensitive for checkpoint inhibition [7]. Therefore, developing new method for PDAC diagnosis and treatment is urgent.

Neoantigen based tumor vaccines have exhibit promising therapeutic effects against multiple solid tumors including PDAC [8–10]. Dendritic cells (DCs) play central roles during antigen presenting processes and are the major focus of tumor vaccines [11]. Mature DCs express co-stimulatory molecules including CD80, CD86, CD40 and CD70, that enables DCs have the capacity to activate T cells and NK cells in tumor immune-surveillance, and directly kill tumor cells [12, 13]. DCs can be pulsed by synthetic peptides that derived from tumor antigens, tumor specific proteins, and even tumor cell lysates, which is a good candidate for adoptive cell therapy [14–16]. However, the efficacy of DC based vaccine against PDAC is limited due to impressive tumor microenvironment and the lack of T cells [17].

IL2, a 15.5 kDa glycoprotein, is mainly secreted by antigen stimulated CD4 + T cell which play a key role in the cancer immunotherapy. Functionally, IL2 can promote the proliferation and differentiation of T cells, enhance the killing activity of NK cells, and induce the generation of cytotoxic T lymphocyte (CTL). IL2 stimulates cells through binding to either a high-affinity IL2 receptor containing the α-, β-, and γ-chains or a low-affinity dimeric receptor consisting of only the β- and γ-chains. However, evidence indicates that IL2 cannot stimulate DCs directly because of the lack of functional IL2 receptor [18]. In the tumor microenvironment, it has been identified that IL2 signals promote the production of cytokines including FLT3L, CSF-2, and TNF from T cells and NK cells, and induces DCs expansion, activation and antigen processing, which resulted in favorable anticancer responses in mice and patients with melanoma [18]. In pancreatic cancer, the combined treatment with allicin and rIL-2 suppresses the xenograft growth and prolonged the survival of the tumor bearing mice through activation of CD4 + T, CD8 + T and NK cell [19]. However, the impact of IL2 on DCs in pancreatic cancer is still unclear. Whether IL2 promotes infiltration and antigen-presenting in pancreatic cancer needs to be investigated.

This study aims to develop a method to improve the DC-based vaccine against PDAC. We analyzed the correlations between IL-2 expression, lymphocyte infiltration, and patient prognosis by using data from TCGA portal. The efficacy of IL-2 in adoptive DC immunotherapy for pancreatic cancer was examined both in vitro and in a xenograft mouse model.

## Materials and methods

### Patients

This study included the clinical information and gene expression profiling data of 178 patients with PDAC from The Cancer Genome Atlas (TCGA). These data were retrieved from https://www.cbioportal.org/ at 19 Feb 2019. The clinical information of enrolled patients in this study were provided in Table 1.

**Table 1:** Clinical characteristics of patients with pancreatic ductal adenocarcinoma in TCGA database

Characteristics	Levels	Overall
Age, n (%)	≤ 65	94 (52.81%)
	> 65	84 (47.19%)
Gender, n (%)	Female	80 (44.94%)
	Male	98 (55.06%)
T stage, n (%)	T1	7 (3.9%)
	T2	24 (13.5%)
	T3	142 (79.8%)
	T4	3 (1.7%)
	Tx and NA	1 (0.55%)
	Not available	1 (0.55%)
N stage, n (%)	N0	49 (27.53%)
	N1	124 (69.66%)
	Nx	4 (2.25%)
	Not available	1 (0.56%)
M stage, n (%)	M0	80 (44.94%)
	M1	4 (2.25%)
	Mx	94 (52.81%)
Pathological stage, n (%)	Stage I	21 (11.79%)
	Stage II	147 (82.58%)
	Stage III	3 (1.69%)
	Stage IV	4 (2.25%)
	Not available	3 (1.69%)

### Immune cell infiltration analysis

The relative abundance of the tumor infiltrating lymphocytes (TIL) in PDAC tissues with different IL2 mRNA expression statues was calculated by using CIBERSORT. The correlation between IL2 and TILs was examined by Spearman’s test.

### Primary T cell and dendritic cell isolation

Human peripheral blood mononuclear cells (PBMCs) from healthy donors were isolated by gradient centrifugation using Ficoll solution (GE healthcare). DCs were isolated by using MagCellect Human Blood Dendritic Cell Isolation Kit (R&D Systems) following the manufacturer’s instructions. Dendritic cells were separated into four groups and plated into the transwell plate. After attach overnight, the DCs were co-cultured with 1 × 10^6^ PBMCs, that were placed in upper chamber, in RPMI-1640 medium with or without 1000IU/mL rIL2. DCs were cultured in medium with or without rIL2 were set as control group. Five days after co-culture, the DCs were subjected to antigen uptake assay.

T cells in PBMCs were stimulated with anti-CD3/CD28 T cell Activator Dynabeads (Invitrogen) for 48 h and then cultured in RPMI-1640 medium (Lonza) with 1000IU/mL rIL2. Five days after co-culture, the cells were cultured in rIL2 free medium, and then co-cultured with activated DCs.

### Cell culture

Human pancreatic adenocarcinoma cell line BxPC-3 and PANC-1, and human immortalized pancreatic ductal cell line hTERT-HPNE were commercially purchased from ATCC. BxPC-3 cells were cultured in RPMI-1640 (ATCC Modification) supplemented with 10% FBS. PANC-1 and hTERT-HPNE cells were cultured in Dulbecco’s modified Eagle’s medium (DMEM) containing 10% fetal calf serum (FCS; Hyclone), 20 mmol/L HEPES, 100 IU/mL penicillin, and 100 µg/mL streptomycin.

### DCs endocytosis analysis

The endocytosis analysis was processed as previous described [20]. Briefly, DCs were incubated with Alexa Fluor 488-dextran(50 µg) at 37℃for 20 min. The endocytosis was halted by rapid cooling of the cells on ice. The cells were washed with cold PBS for three times and then subjected to flow cytometry. Results were analyzed by Flowjo v10.0.7, and the mean fluorescence intensity (MFI) represented the amount of incorporated tracer.

### Antigen pulsing of DCs

DCs were incubated with freeze-thawed tumor cell lysates at a ratio 1:3(DCs: tumor cells). After 18 h incubation, DCs were harvested by gently pipette, and washed with PBS twice and then resuspended by RPMI-1640 medium for further studies.

### T cell activation

T cells were co-cultured with tumor cell lysate pulsed DCs at a responder-to-stimulator ratio of 20:1. Five days after co-culture, the cells were harvested and subjected to in vitro killing assay and in vivo treatment for xenograft mouse model.

### Immunohistochemistry (IHC)

Paraffin-embedded sections were firstly deparaffinized and then incubated with rabbit anti-CD3 or anti-CD11c monoclonal antibody (Cell Signaling Technology) at 4 °C overnight. After three times wash by TBST, the sections were incubated with HRP conjugated goat anti-rabbit secondary antibody (Cell Signaling Technology). The sections were washed by TBST three times, and the signals were detected using DAB Substrate kit following the manufacturer’s instructions. Images were obtained using microscopy.

### Flow cytometry

Cells were washed once with flow cytometry buffer, and then stained with dilutions of various combinations of primary antibodies to cell surface marker including CD3, CD4, CD8, CD11c, CD14, CD19, CD40, CD80, CD83, CD86, CCR7, CD123, BDCA1, BDCA2, BDCA3 and PD1. After that, cells were washed with PBS and then subjected to flow cytometry analysis. For intracellular staining, cells were first permeabilized with Intracellular Staining Permeabilization Wash Buffer (BioLegend, Biotechnology Co. Ltd., USA), and then incubated with primary antibodies targeting Foxp3, IFN-γ or IL-4. Cells were washed with PBS and then subjected to flow cytometry analysis.

For apoptosis analysis, DCs were collected after different treatments and then incubated with Annexin V-FITC and propidium iodide (PI). The apoptotic cells were determined by flow cytometry and the results were analyzed by using FlowJo software (v10.0.7) (Tree Star, Inc., Ashland, OR, USA).

### Human IFN-γ, TGF-β, VEGFA and IL-10 enzyme-linked immunosorbent assay (ELISA)

The cell culture supernatants were collected for cytokines detection. The concentrations of IFN-γ, TGF-β, VEGFA and IL-10 were measured by using the Human IFN-γ DuoSet ELISA kit (R&D system), Human TGF-beta 1 DuoSet ELISA kit (R&D system), Human VEGF DuoSet ELISA kit (R&D) and Human IL-10 DuoSet ELISA kit (R&D systme) respectively, following the manufacturer’s instructions.

### In vitro killing assay

Tumor cells were seeded into 96-well plate and allow attach overnight. T cells were then seeded into the plate at a E (effector cells): T(target cells) ratio of 5:1. The continuous tumor cell death was evaluated every hour for 25 h by using xCELLigence impedance-based system. The normalized cell index was determined by measuring the impedance of current across the transistor plate caused by tumor cell adherence.

### Xenograft mouse model

Twenty to eight-week-old NOD-SCID mice were purchased from Charles River Laboratories. The left flank of female NOD-SCID mice were injected with 2 × 10^5^ BxPC-3 cells to construct the xenograft mouse model. A total of 1 × 10^5^ of the co-cultured DCs and T cells were injected through the tail vein at 3 and 6 days after tumor cell injection. Tumors were measured every 7 days with a caliper and the tumor volume (mm^3^) was calculated as 0.5 × length × width^2^. Mice were euthanized when the diameter of the tumor reached 20 mm. All experiments including mice were approved by the Shanghai Ruijin Hospital Institutional Review Board.

### Synthetic peptides

The peptide KRAS-G12D_1 − 23_ (MTEYKLVVVGADGVGKSALTIQLIQ) was synthesized by standard solid-phase synthetic peptide chemistry and purified using reversed-phase high-performance liquid chromatography (HPLC) (GenScript). DCs were incubated with the neoantigen peptides (50 µg/ml) for 18 h, and then harvested for T cell activation.

### Statistical analyses

Data were analyzed using analysis was conducted using R (version 4.2.2) via RStudio (Desktop version, 2022.12.0 + 353). One-way ANOVA was used as multiple comparisons test to analyze datasets containing more than two groups. The survival of patients and mice was analyzed using the Kaplan-Meier method with the log-rank test. The cutoff point was calculated by using surv_cutpoint function of survminer package. The cutoff point for IL12 mRNA expression is 0.1373. *P* < 0.05 was considered to indicate statistical significance.

## Results

### Prognostic value of IL2 in pancreatic cancer

To assess the role of IL-2 in pancreatic ductal adenocarcinoma (PDAC), we compared the levels of IL-2 in tumors from 178 PDAC patients obtained from the TCGA portal with 171 normal pancreases from the GTEx portal. The analysis revealed that IL-2 levels were relatively lower in normal pancreases, although the difference was not statistically significant (Fig. S1A). Subsequently, we generated a Kaplan-Meier survival curve using TCGA data, which demonstrated that patients with high expression of IL-2 mRNA in their tumors had a higher overall survival rate compared to those with low expression of IL-2 mRNA (*p* = 0.018, Fig. S1B). In a multivariate Cox analysis that considered age, sex, tumor grade, and tumor stage as confounding factors, patients with high expression of IL-2 mRNA had a hazard ratio (HR) for disease mortality of 0.52 (95% CI 0.33–0.81; *p* = 0.004; Fig. S1C). These findings indicate that high expression of IL-2 mRNA in PDAC tumors is associated with improved overall survival and a reduced risk of disease mortality.

### Association of IL2 with the tumor immune microenvironment

To investigate the immune microenvironment in relation to IL-2 expression, we employed CIBERSORT analysis to examine the relative proportions of different immune cell types [21]. Our analysis revealed significant associations between IL-2 mRNA expression and specific immune cell populations. We observed a positive correlation between IL-2 expression and the proportions of CD8 + T cells, activated NK cells, naïve B cells, and resting myeloid dendritic cells (DCs). In contrast, IL-2 expression exhibited a negative correlation with M0 macrophages, suggesting that IL-2 may play a role in promoting the migration of T cells, NK cells, B cells, and dendritic cells within the tumor microenvironment (Fig. S2).

Comparison of the expression of LM22 gene signature identified by CIBERSORT between high and low IL-2 mRNA expression groups in TCGA cohort.

### IL2 promotes DCs proliferation and antigen uptake indirectly

**Fig. 1:**
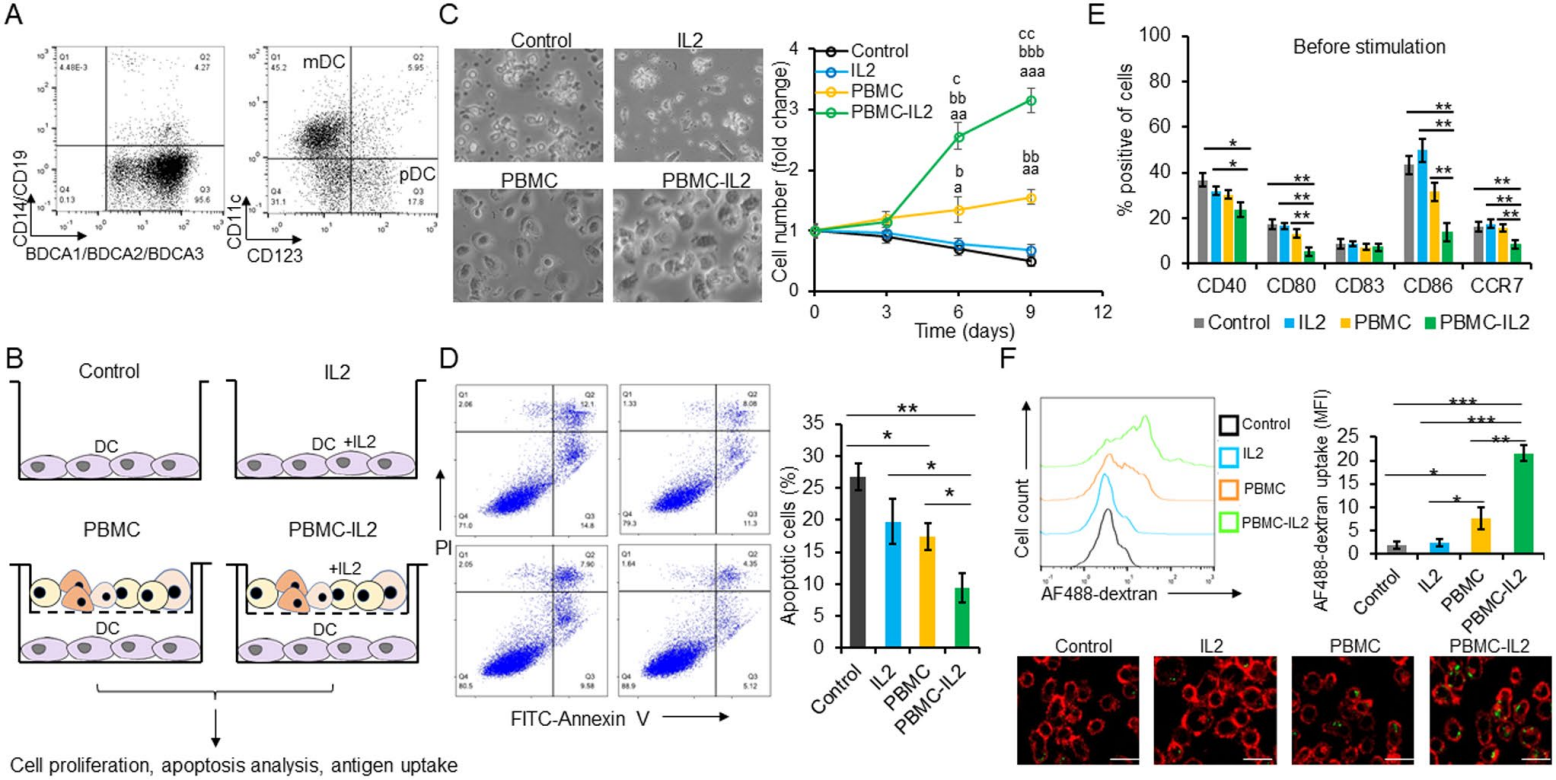
IL2 promotes DCs proliferation and antigen uptake when co-cultured with PBMC. (**A**) Isolated DCs were stained with antibodies targeting cell surface markers and then subjected to flow cytometry analysis. (**B**) Schematic diagram indicates the four different in vitro culture conditions for DCs. (**C**) The numbers of DCs were calculated every 3 days. (**D**) The cell apoptosis was examined by flow cytometry 6 days after culture. (**E**) DCs were incubated with antibodies targeting maturation markers including CD40, CD80, CD83, CD86 and CCR7. After washing by PBS, the cells were subjected to flow cytometry analysis. (**F**) Antigen uptake assay. The antigen positive cells were quantified by flow cytometry. The morphology of cells was captured by confocal microscopy. bar = 50 μm

DCs can be pulsed by tumor antigens and used for tumor adoptive cell therapy [14–16]. However, due to the lack of functional IL-2 receptors, evidence suggests that IL-2 cannot directly stimulate DCs [18]. In order to investigate the role of IL-2 in DCs for tumor adoptive cell therapy, we isolated DCs from human peripheral blood. As shown in Fig. 1A, the purity of DCs was more than 95%, and the myeloid DCs is the major proportion of the isolated DCs. DCs were cultured under four different conditions (Fig. 1B). As shown in Fig. 1C, IL2 could not directly promote the proliferation of DCs. However, when co-cultured with PBMCs, the cell number of DCs were significantly upregulated in the cell culture condition with IL2 (Fig. 1C). Meanwhile, IL-2 demonstrated the ability to inhibit DC apoptosis only when DCs were co-cultured with PBMCs (Fig. 1D). Additionally, DCs cultured with IL-2 and PBMCs expressed less maturation markers including CD40, CD80, CD86 and CCR7(Fig. 1E), suggesting an immature status.

To examine the impact of IL-2 treatment on antigen uptake, DCs were initially cultured in a medium containing GM-CSF. Two days before the antigen uptake assay, DCs were then cultured under different conditions shown in Fig. 1B, without extra GM-CSF addition. As shown in Fig. 1F, IL2 significantly promoted DCs antigen uptake when co-cultured with PBMCs. These results indicated that IL2 may induce cytokines release from PBMCs and promote DCs proliferation and antigen uptake.

### IL-2 promotes the efficacy of DC based tumor vaccine in vitro

**Fig. 2:**
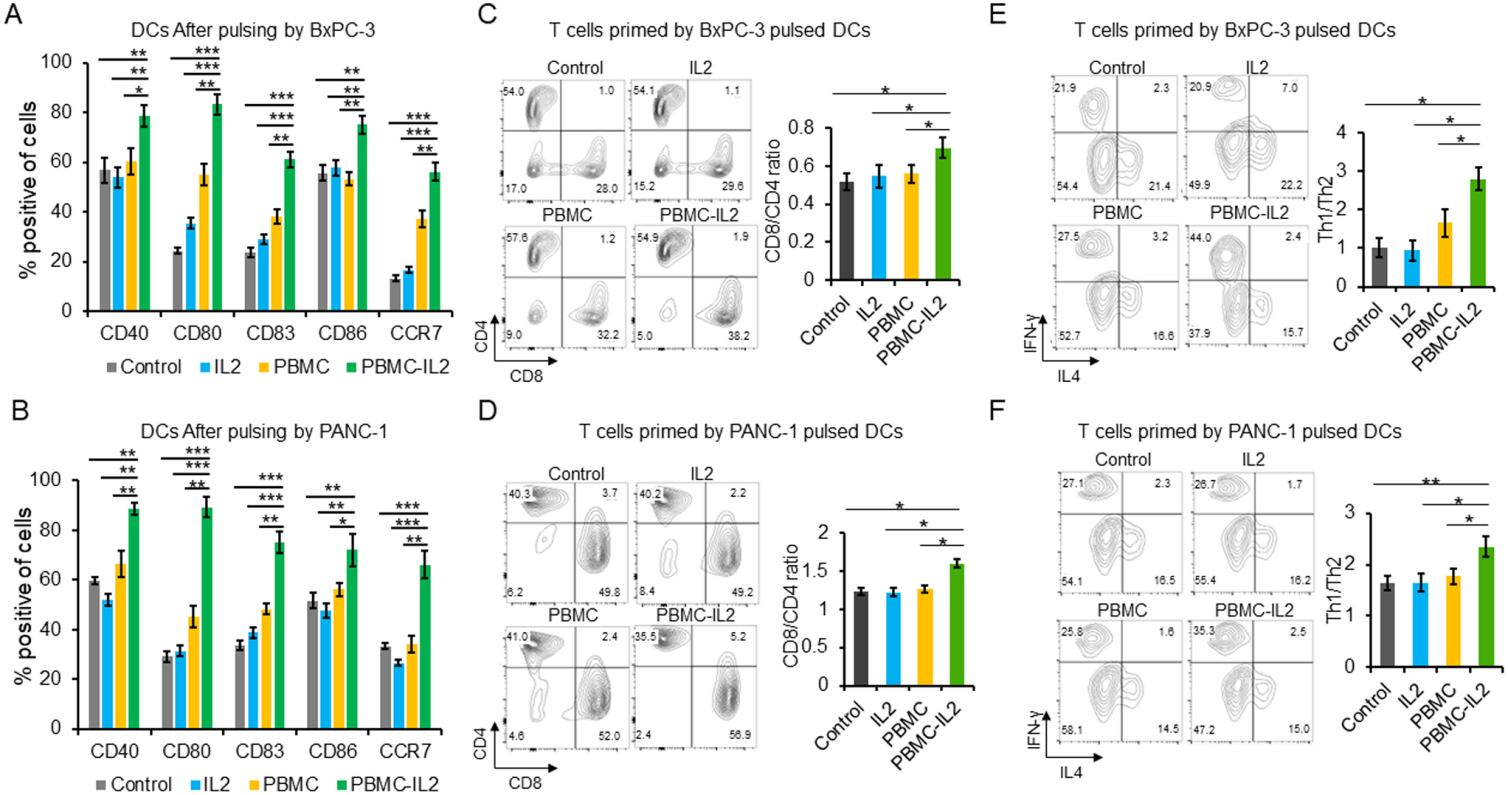
PBMC and IL-2 promote the maturation of DCs and expansion of CD8 + T cells and Th1 cells. DCs were pulsed with freeze-thawed BxPC-3 (**A**) or PANC-1(**B**) cell lysates for 18 h. The cells surface levels of CD40, CD80, CD83, CD86 and CCR7 were detected by flow cytometry. T cells were activated by different groups of DCs for 5 days. The cells surface CD4, CD8 (**C** and **D**), and intracellular IFN-γ and IL-4 (**E** and **F**) were detected by flow cytometry

**Fig. 3:**
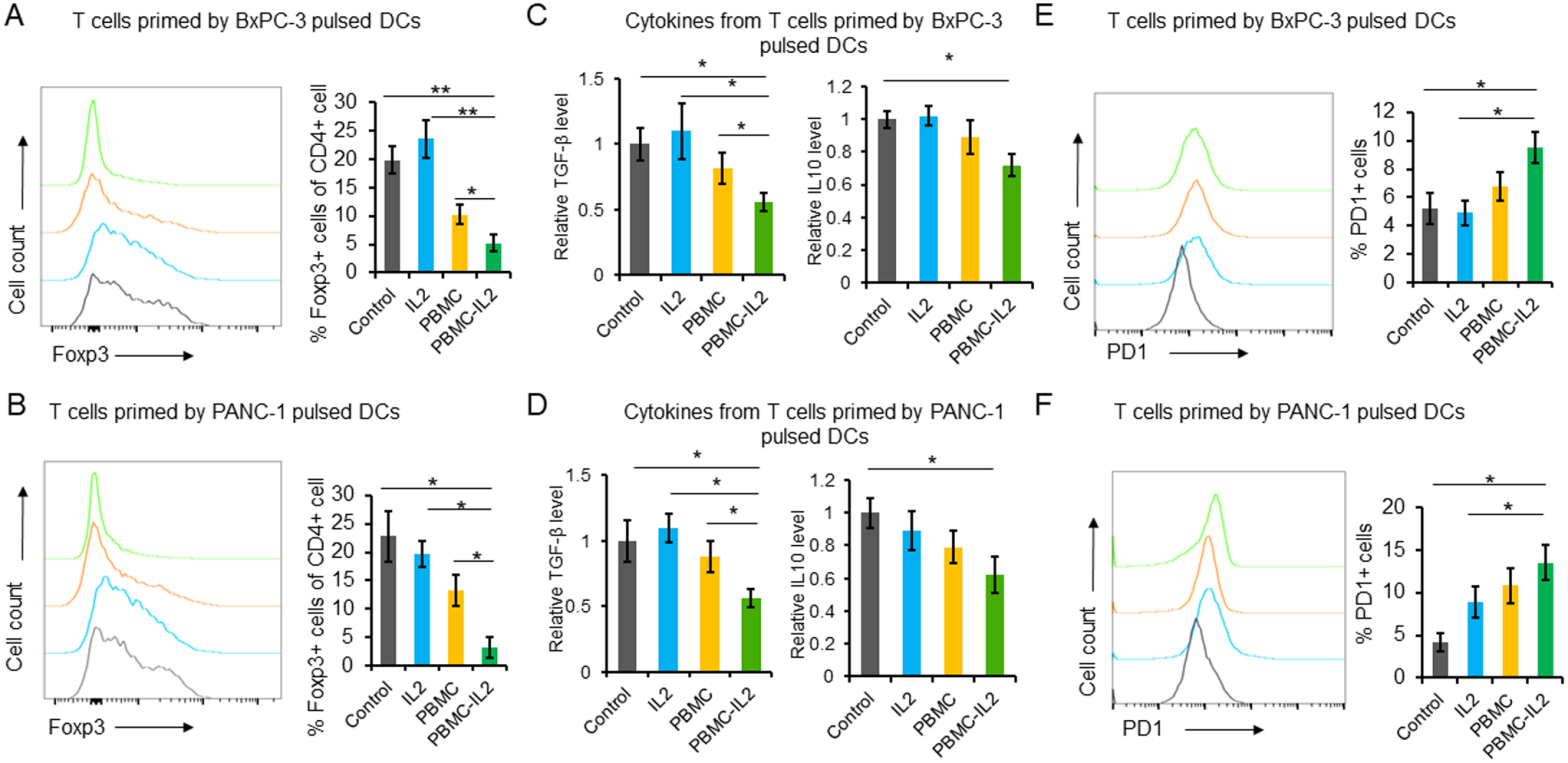
PBMC and IL-2 inhibit the expansion of Tregs, the production of TGF-β and IL-10 but upregulate PD1 expression. (**A**) T cells were activated by different groups of DCs for 5 days and the intracellular Foxp3 was detected by flow cytometry. (**B**) The concentrations of TGF-β and IL-10 in the medium were examined by ELISA. (**C**) The cell surface PD-1 level was detected by flow cytometry

**Fig. 4:**
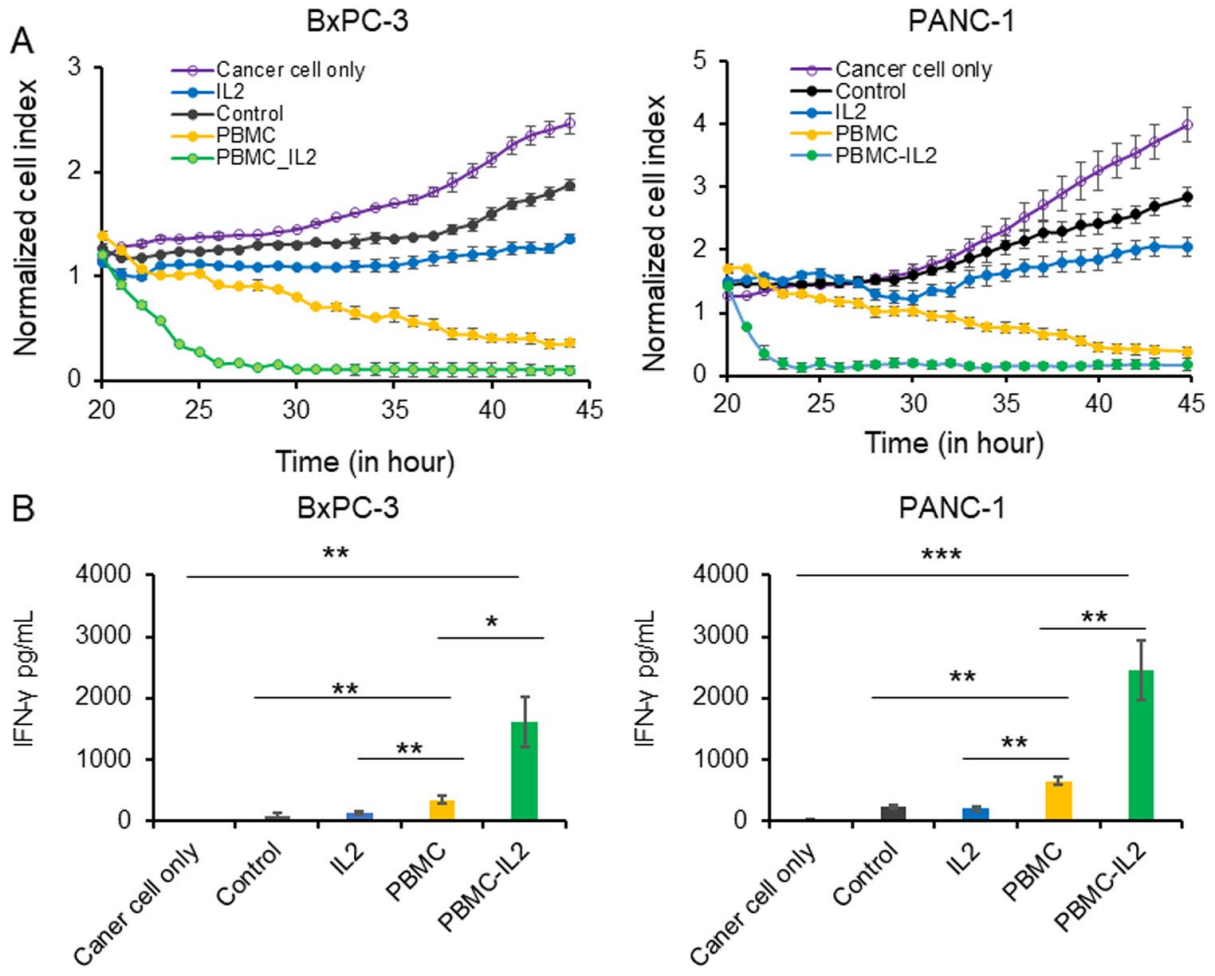
PBMC and IL-2 promote the efficacy of DC based tumor vaccine in vitro. (**A**) in vitro killing assay. Tumor cells were seeded into 96-well plates and allow attach overnight. T cells were then seeded into the plate at a ratio of 5:1. The continuous tumor cell death was evaluated every hour for 25 h by using xCELLigence impedance-based system. (**B**) ELISA. The IFN-γ level in the supernatants was examined by ELISA

To investigate the impact of IL-2 on adoptive DC therapy for pancreatic cancer, DCs were cultured under four different conditions and then pulsed with freeze-thawed BxPC-3 or PANC-1 cells. We observed that DCs pulsed by tumor cell lysates in PBMC-IL-2 group expressed increased maturation markers (Fig. 2A and B). Subsequently, T cells were co-cultured with DCs from different groups respectively for five days. T cells that were activated by DCs from PBMC-IL2 group had increased CD8/CD4 (Fig. 2C and D) and Th1/Th2(Fig. 2E and F) ratios. Meanwhile, they had a reduced proportion of regulatory T cells (Tregs) (Fig. 3A and B), secreted reduced TGF-β and IL-10 (Fig. 3C and D), but expressed increased PD-1 (Fig. 3E and F). Results of in vitro killing assay indicated that all the four groups of T cells could repress the tumor cell growth in vitro, especially the T cells vaccinated by DCs that co-cultured with PBMC and IL-2 (Fig. 4A). T cells primed by the DCs that co-cultured with PBMCs in the medium containing IL2 had the strongest repression effect and secreted the highest level of IFN-γ (Fig. 4A and B).

To examine whether the DC-primed T cells targeted tumor cells specifically, we used immortalized human pancreatic ductal cell line hTERT-HPNE as negative control. DC-primed T cells dramatically repressed tumor cell growth and secreted the highest levels of IFN-γ, indicating that these T cells could target tumor cells specifically (Fig. S3).

### IL-2 promotes the efficacy of DC based tumor vaccine in xenograft mouse model

**Fig. 5:**
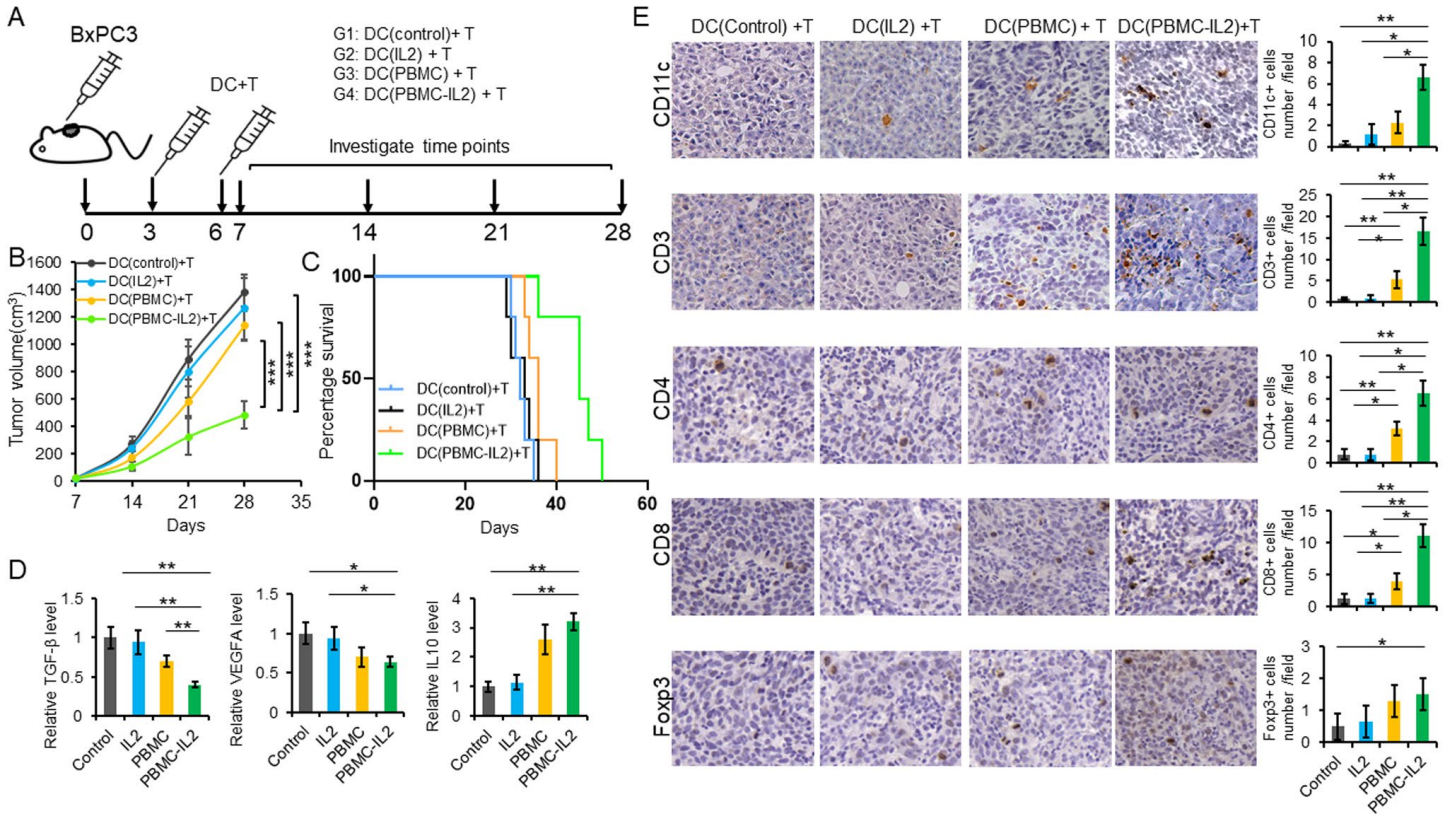
IL-2 promotes the efficacy of DC based tumor vaccine in xenograft mouse model. (**A**) Schematic diagram of the construction and treatment process of BxPC3 mouse model. (**B**) Tumor volumes were monitored every 7 days. (**C**) Kaplan-Meier survival analysis. (**D**) The levels of TGF-β, VEGFA and IL-10 in the tumor lysates were detected by ELISA. (**E**) Immunohistochemistry. The T cell markers CD3, CD4, CD8, Treg marker Foxp3, and DCs marker CD11c were detected by immunohistochemistry

To explore whether IL-2 promotes the production of sustained T cell immunity to pancreatic cancer cells in vivo, xenograft mouse model was generated via subcutaneous BxPC3 cells injection. The mice were randomly divided into four groups depending on their following treatment: group1, DC (control); group2, DC(IL2) + T; group3, DC(PBMC) + T; group4, DC(PBMC-IL2) + T. DCs and T cells mixture was administered through the tail vein at 3 and 6 days after tumor cell injection (Fig. 5A). As depicted in Fig. 5B, tumor growth was significantly inhibited by T cells activated by DCs co-cultured with PBMCs in the presence of IL-2. Moreover, group 4 mice exhibited the longest survival time (Fig. 5C). Group 4 tumors had significantly reduced TGF-β, VEGFA but increased IL-10 (Fig. 5D). Meanwhile, group 4 tumors had increased DCs, total T cells, CD4 + T cells and CD8 + T cells (Fig. 5E). The numbers of Tregs were increased in group 4 tumors when compared with group 1, but they are not significantly different among group 2, 3 and 4 (Fig. 5E).

Furthermore, we compared the efficacy of DCs obtained through the traditional method (culturing DCs in a medium containing GM-CSF and IL-4) with that of DCs cultured with PBMCs and IL-2. As shown in Fig. S4 A and B, the efficacy of these two methods did not differ significantly, as evidenced by similar tumor growth and mouse survival rates. However, DCs cultured with PBMCs, and IL-2 exhibited higher levels of infiltration into the tumors (Fig. S4C), potentially facilitating sustained T cell immunity that exhibited reduced Tregs (Fig. S4C) and reduced IL-10 (Fig. S4D).

### IL-2 driven the generation of KRAS G12D specific cytotoxic T cells

**Fig. 6:**
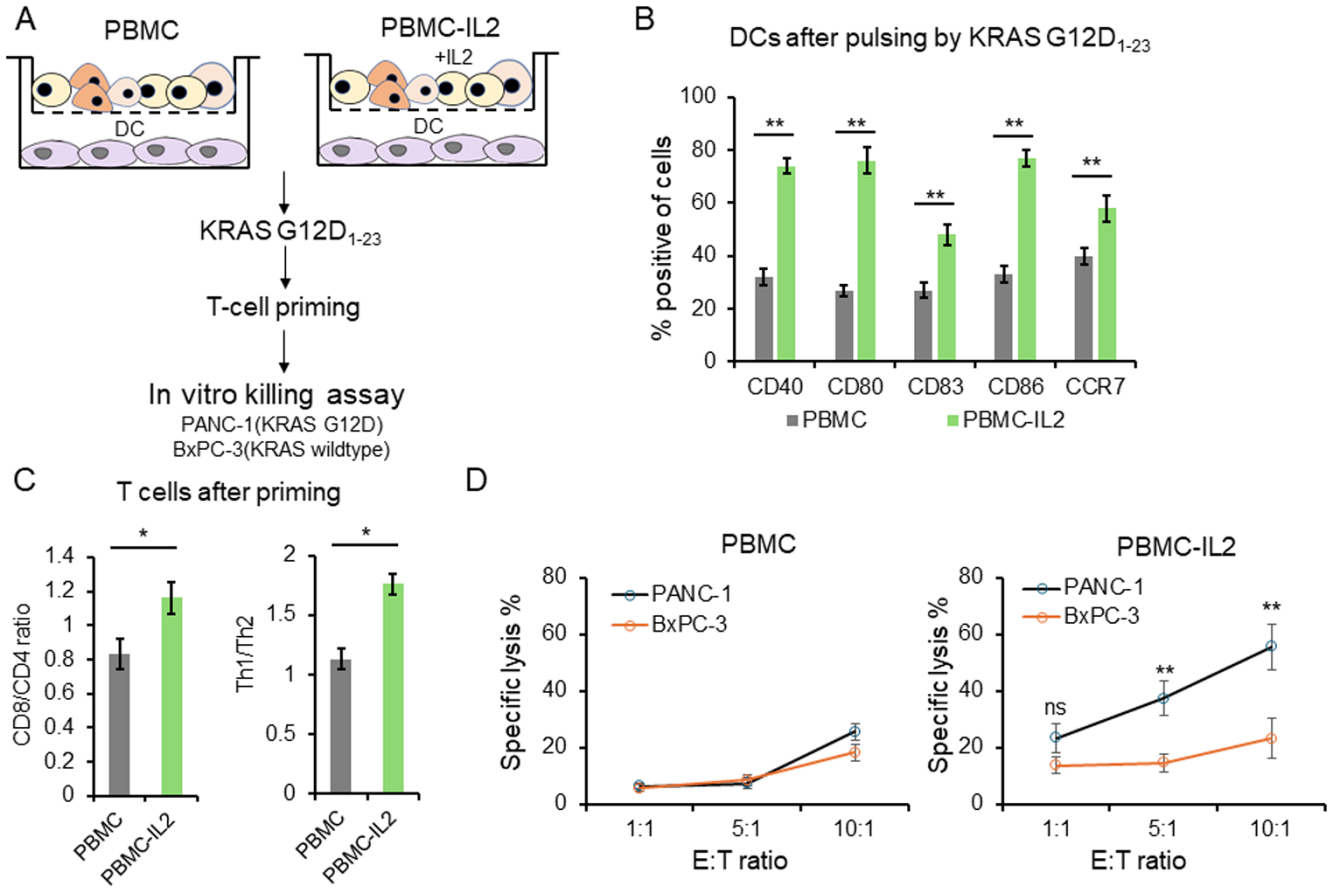
IL-2 driven the generation of KRAS G12D specific cytotoxic T cells. (**A**)Schematic diagram for DC culture, stimulation, T-cell priming and in vitro killing assay. (**B**) DCs were pulsed with KRAS G12D_1 − 23_ peptide for 18 h. The cells surface levels of CD40, CD80, CD83, CD86 and CCR7 were detected by flow cytometry. (**C**) T cells were activated by DCs for 5 days. The CD8/CD4 ratio and Th1/Th2 ratio were determined by flow cytometry analysis by detecting cells surface CD4, CD8, and intracellular IFN-γ and IL-4. (**D**) T cells were co-cultured with GFP expressing PANC-1 or BxPC-3 cells at different E:T ratio for 5 h. The remaining cancer cells were counted using flow cytometry

KRAS is the most frequently mutated oncogene in pan-cancer, and the position 12 glycine is the mutation hot spot [22]. To explore whether IL-2 can promote the generation of cytotoxic T cells targeting cancer cells carrying mutant KARS, DCs were pulsed with KRAS G12D_1 − 23_, and then used for T cell priming. PANC-1(KRAS G12D mutant) and BxPC-3(KRAS wildtype) cells were used for T cell targets to examine the specificity of cytotoxicity (Fig. 6A). DCs co-cultured with PBMC and IL-2 expressed increased maturation markers after KRAS G12D_1 − 23_ stimulation (Fig. 6B). Meanwhile, the primed T cells had an increased CD8/CD4 ratio and Th1/Th2 ratio (Fig. 6C). T cells primed by KRAS G12D_1 − 23_ pulsed DCs that cultured with PBMC had a 5.7–18.2% inhibition for BxPC-3 and 6.3–25.5% inhibition for PANC-1 cells at E: T from 1:1 to 10:1(Fig. 6D left panel). T cells primed by KRAS G12D_1 − 23_ pulsed DCs that cultured with PBMC-IL-2 killed 37.3% PANC-1 cells compared with 14.5% for BxPC-3 cells at E: T of 5:1 and killed 55.5% PANC-1 cells compared with 23.2% BxPC-3 cells at E: T of 10:1 (Fig. 6D right panel). These results indicate that IL-2 drove the generation of KRAS G12D specific cytotoxic T cells.

## Discussion

IL-2 was initially discovered in the culture supernatants of activated T cells and subsequently utilized for the expansion of patients’ T cells and NK cells [23, 24]. In the 1990s, IL-2 received approval from the US Food and Drug Administration (FDA) for the treatment of metastatic renal cell carcinoma and metastatic melanoma [25, 26]. Previous studies have indicated that preoperative IL-2 immunotherapy increased the 2-year survival rate in patients with pancreatic cancer [27]. However, the clinical use of IL-2 is limited due to severe toxicities and off-target effects [25, 26]. In this study, we investigated the mRNA levels of IL-2 in 178 PDAC patients and discovered that IL-2 could serve as an independent prognostic biomarker. We also analyzed the immune contexture in different IL2 mRNA expression status and noticed that higher IL-2 mRNA relates to increased proportion of tumor infiltrating lymphocytes (TILs), especially CD8 + T cells, naïve B cells and NK cells. This may explain the patient favorable role of IL2.

It is identified that DCs do not express functional IL-2 receptors, but IL-2 signals could promote the production of cytokines from T cells and NK cells, that induces DCs expansion, activation and antigen processing [18]. As the main antigen presenting cells, DCs have been used as a typical vector for tumor antigen delivery and priming antitumor immunity. We noticed that higher IL-2 level in PDAC relates to increased resting dendritic cells. However, the function of IL-2 on DCs based tumor vaccine is unclear. In this study, we cultured DCs in four different conditions and confirmed that IL-2 alone could not promote the expansion and antigen presenting of DCs. When PBMCs exist, DCs based tumor vaccine could prime the antitumor immunity of T cells which is consistent with the report of Raeber et al [18]. Meanwhile, we observed DCs cultured with PBMCs, and IL-2 promoted anti-tumor capacity by producing increased CD8 + cells and Th1 cells but reduced Tregs. The upregulation of PD1 positive T cells suggests a potential synergistic effect with anti-PD-1 antibodies. Meanwhile, in the tumors, TGF-β and VEGFA levels were reduced by IL-10 level was increased in PBMC-IL-2 group. That may be the result of multiple origin of TGF-β and VEGFA, but IL-10 is mainly produced by tumor infiltrated T cells. These results also suggest that anti-IL-10 antibody may improve the efficacy of DC based vaccine. Furthermore, DCs themselves could also infiltrated into the tumors indicating that IL-2 treatment could promote a sustained T cell immunity to pancreatic cancer not only by generating tumor specific T cells but also upregulating DCs infiltration. The mechanism may be related to activated cell viability and migration capacity that need to be further investigated.

Currently, a great interest has been paid to DCs because their potential for developing antitumor vaccines. Emerging evidence has confirmed that DC based tumor vaccines can induce the generation of tumor antigen specific cytotoxic T cells. However, the efficacy of DC based vaccine against PDAC is limited due to impressive tumor microenvironment and the lack of T cells [17]. Meanwhile, single antigen approaches may demonstrate limited therapeutic efficacy due to the presence of tumor heterogeneity and the potential for immune escape. In this study, we pulsed DCs by using tumor cell lysates to overcome this challenge. We observed that T cells primed by DCs could specifically target tumor cells. Although the growth of hTERT-HPNE was partially repressed by T cells, that may be the effect of T cell secreted cytokines. However, cancer cell lysates contain multiple normal protein generated peptides, which have the potential to induce the production of T cells targeting non-tumor cells. Therefore, DC based vaccines targeting multiple tumor antigens seem to be more promising and need to be extensively investigated [28].

One of the typical methods for in vitro DCs generation is culturing them from PBMC in the presence of IL-4 and GM-CSF [29, 30]. In our study, we compared the effectiveness of DC-based adoptive cell therapy against PDAC using different culturing conditions and found that coupling IL2 with PBMC could yield similar or even more potent DCs. Although the efficacy against PDAC did not show significant differences compared to the traditional method, we observed that more DCs cultured with PBMCs infiltrated into the tumors, potentially facilitating sustained antitumor immunity. Meanwhile, tumors treated by T cells that primed by PBMC- IL2 co-cultured DCs had reduced Tregs number and IL-10 level, suggesting an improved microenvironment. However, it is important to note that DCs consist of various subtypes, and the cytokines used for in vitro differentiation can impact downstream T cell responses. Therefore, a detailed IL2-PBMC functions on each subtype of DCs needs to be dissected for improving the efficacy of DCs based vaccines. Additionally, it is recommended to use tumor-specific antigens instead of tumor cell lysates to enhance specificity and reduce potential toxicity in DC-based vaccines.

In summary, this study highlights the favorable clinical diagnostic value of IL-2 in patients with PDAC for the first time and confirms that IL-2 can enhance the efficacy of DC-based tumor vaccines both in vitro and in vivo, providing a promising new approach for PDAC immunotherapy.

### Electronic supplementary material

Below is the link to the electronic supplementary material.

Supplementary Material 1

## Data Availability

All data generated or analyzed during this study are included in this published article.
