# Supplementary material for: IL-2 promotes expansion and intratumoral accumulation of tumor infiltrating dendritic cells in pancreatic cancer
Source: Cancer Immunol Immunother. 2024 Mar 30;73(5):84. doi: 10.1007/s00262-024-03669-7 (PMC10981618; doi:10.1007/s00262-024-03669-7)
Supplement: Supplementary file 1 — Supplementary Material 1 [file 262_2024_3669_MOESM1_ESM.docx]

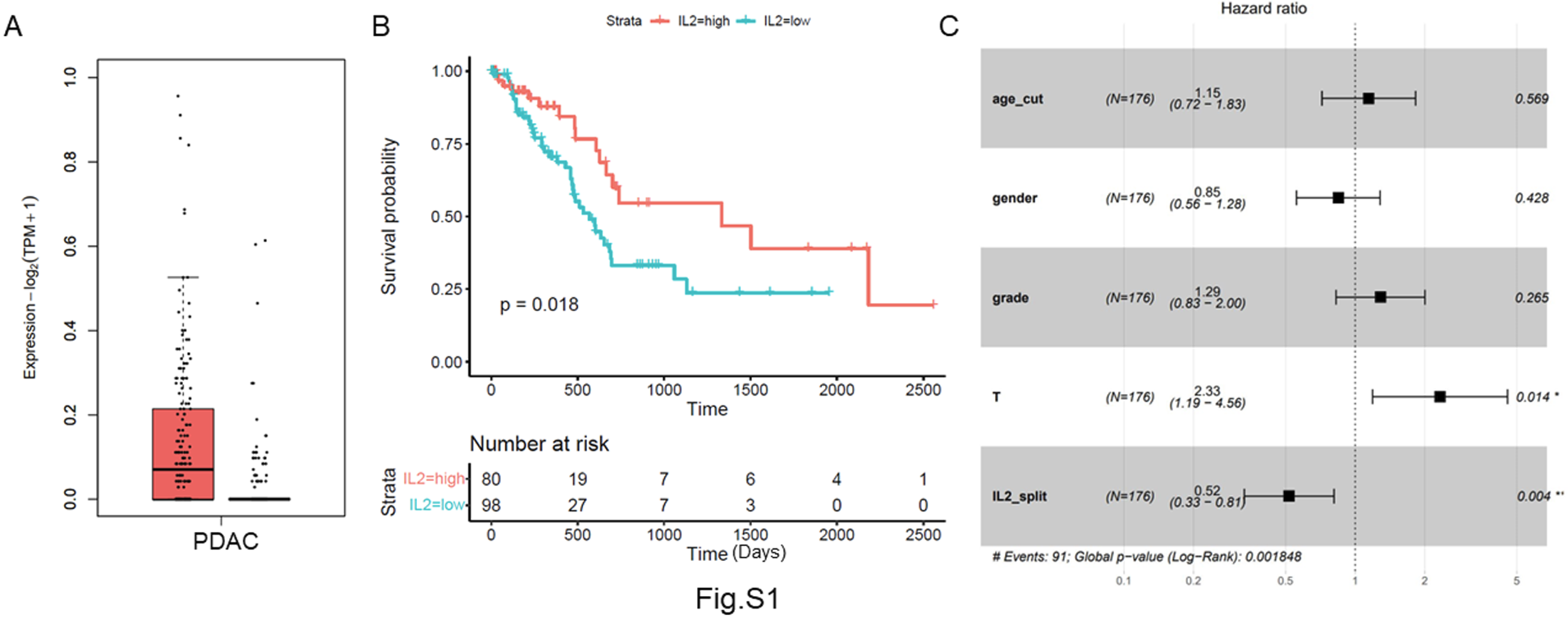


**Fig. S1. Prognostic value of IL-2 in pancreatic cancer**

(A) The IL-2 level in 178 PDAC samples (TCGA) and 171 normal pancreases (GTEx) were analyzed by using online bioinformatic tool GEPIA2. (B) IL-2 mRNA levels for overall survival in the TCGA. (C) Multivariate cox analysis for IL-2 mRNA expression and clinicopathological variables.


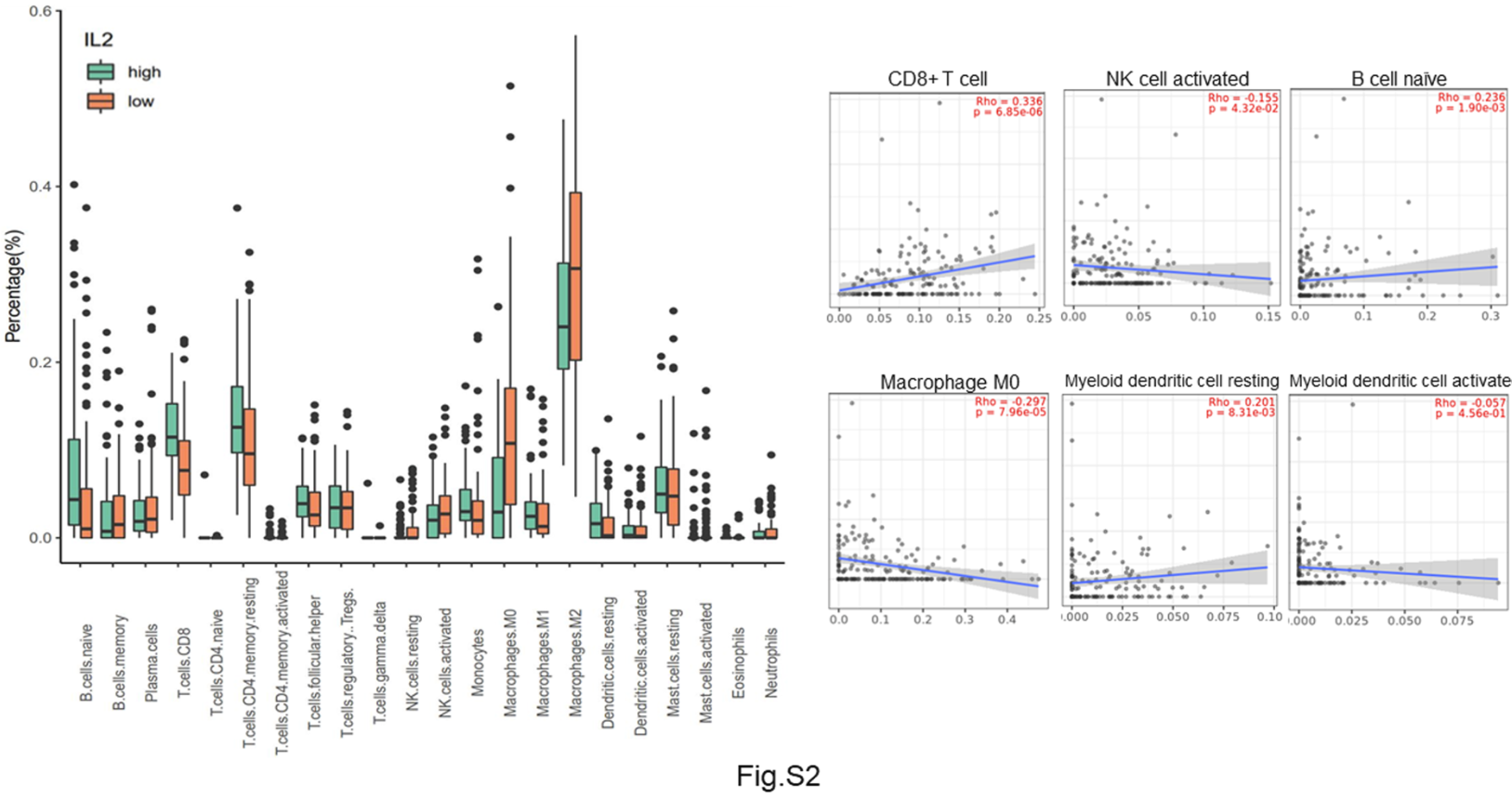


**Fig. S2. Comparison of the infiltration of various immunocytes between high and low IL-2 groups.**

Comparison of the expression of LM22 gene signature identified by CIBERSORT between high and low IL-2 mRNA expression groups in TCGA cohort.


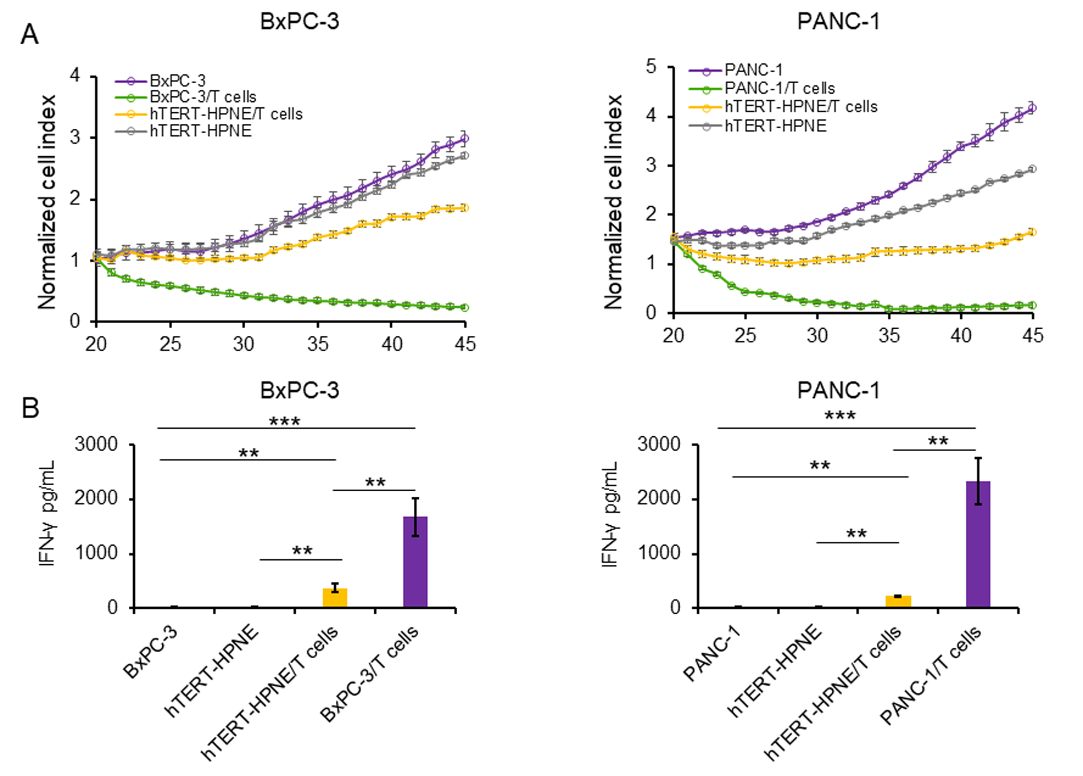


**Fig. S3. T cells primed by DC vaccines targets tumor cells specifically.**

(A) in vitro killing assay. Tumor cells were seeded into 96-well plates and allowed attach overnight. T cells were then seeded into the plate at a ratio of 5:1. The continuous tumor cell death was evaluated every hour for 25 hours by using xCELLigence impedance-based system. (B) ELISA. The IFN-γ level in the supernatants was examined by ELISA.


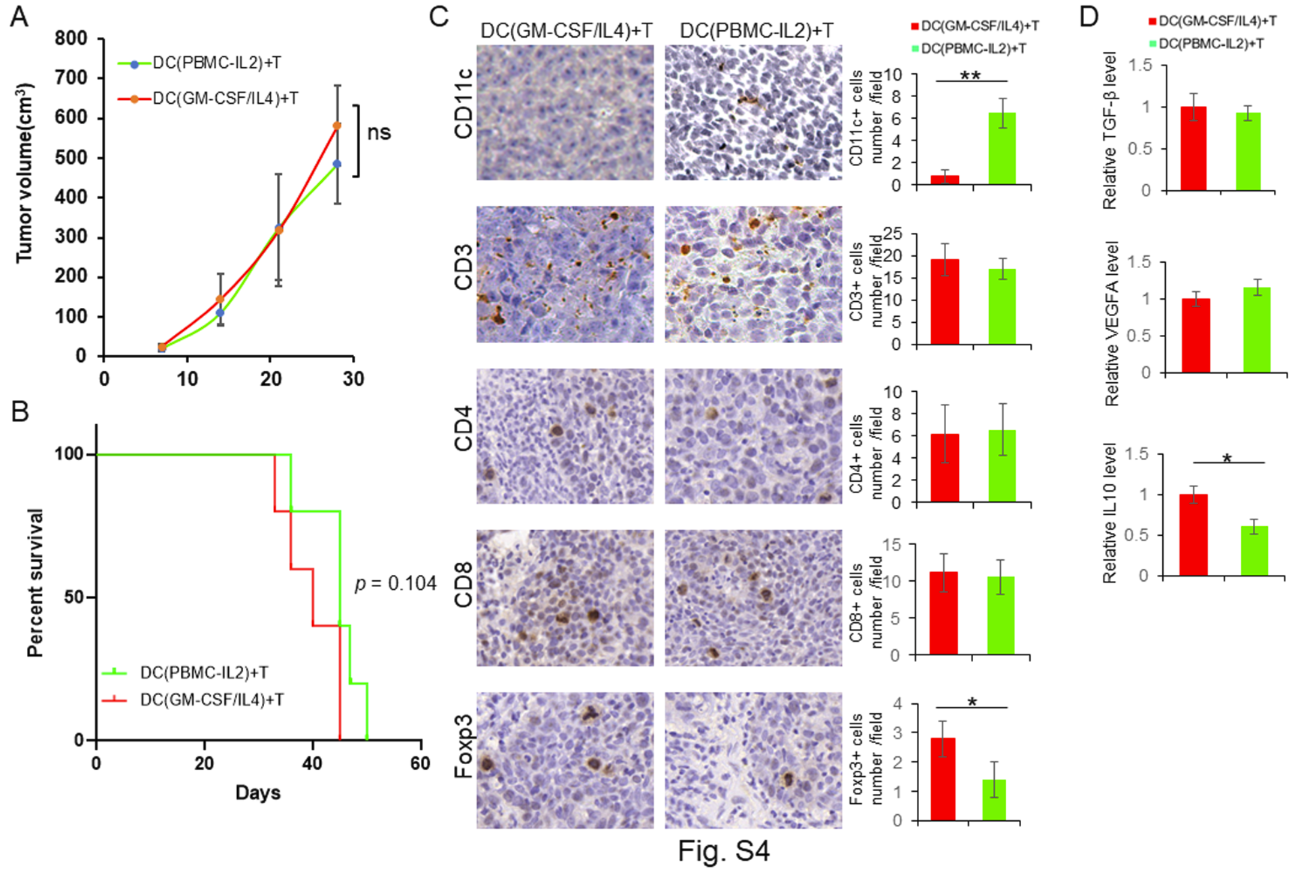


**Fig. S4. Compared the efficacy of PBMC-IL-2 cultured DCs vaccine and a typical GM-CSF/IL-4 cultured DCs vaccine.**

BxPC3 mouse model was generated by subcutaneous injection. The mixture of DCs and T cells was administered via the tail vein. (A)Tumor volumes were monitored every 7 days. (B) Kaplan-Meier survival analysis. (C) Immunohistochemistry. The T cell marker CD3, CD4, CD8, Treg marker Foxp3, and DCs marker CD11c were detected by immunohistochemistry.
